# Exploring the combined effect of optimally controlled chemo-stem cell therapy on a fractional-order cancer model

**DOI:** 10.1371/journal.pone.0311822

**Published:** 2025-02-13

**Authors:** Kaushik Dehingia, Sana Abdulkream Alharbi, Awatif Jahman Alqarni, Mounirah Areshi, Mona Alsulami, Reima Daher Alsemiry, Reem Allogmany, Homan Emadifar, Mati ur Rahman

**Affiliations:** 1 Department of Mathematics, Sonari College, Sonari, Assam, India; 2 Mathematics Research Center, Near East University, TRNC, Nicosia, Turkey; 3 Department of Mathematics & Statistics, Taibah University, Yanbu, Al-madinah Al-munawarah, Saudi Arabia; 4 Department of Mathematics, College of Science, University of Bisha, Bisha, Saudi Arabia; 5 Department of Mathematics, Faculty of Science, University of Tabuk, Tabuk, Saudi Arabia; 6 Department of Mathematics, Faculty of Science, University of Jeddah, Jeddah, Saudi Arabia; 7 Department of Mathematics, Faculty of Science, Taibah University, Al-madinah Al-munawarah, Saudi Arabia; 8 Department of Mathematics, Saveetha School of Engineering, Saveetha Institute of Medical and Technical Sciences, Chennai, Tamil Nadu, India; 9 MEU Research Unit, Middle East University, Amman, Jordan; 10 Department of Mathematics, Hamedan Branch, Islamic Azad University, Hamedan, Iran; 11 School of Mathematical Sciences, Jiangsu University, Zhenjiang, Jiangsu, P.R.China; 12 Department of Computer Science and Mathematics, Lebanese American University, Beirut, Lebanon; Universiti Tun Hussein Onn Malaysia, MALAYSIA

## Abstract

This study aims to investigate the effect of fractional order on a novel cancer treatment model in the Caputo sense with chemotherapy and stem cell therapy. The existence of positive solutions, equilibria, and linear stability of the model are examined. The Ulam-Hyers stability of the system is investigated. An optimal treatment schedule is developed to obtain the combined effect of chemotherapy and stem cell therapy on the model. The analytical results are verified through numerical examples. It has been observed that stem cell therapy and effector cells alone cannot eradicate tumor cells effectively. However, in the presence of optimally controlled chemotherapy, there is an effective reduction in the population of tumor cells, while the populations of normal and effector cells progressively grow over time.

## Introduction

Cancer is one of the most frequently encountered diseases, and it causes around 200 different diseases with different characteristics. It is a multi-staged disease that occurs due to changes in the DNA mutations of abnormal cells. During the evolution and progression stages of cancer, many cells, cytokines, and chemokines of the immune system are responsible for protecting other healthy tissues from the invasion of cancerous cells. At these stages, the tumor-immune interaction is very complex and chaotic. The mathematical modelling study helps the researcher to predict the complex and chaotic behavior of the tumor-immune system [1–4], and it also helps to investigate the efficacy of different treatment strategies that are applied to eradicate cancer cells [5–10].

Fractional calculus and its application are now a growing research area in various fields such as applied mathematics [11, 12], physics [13], biology [14–16], medical science [17], epidemiology [18–20], etc. due to the memory effect of the fractional operator. Thus, several researchers have applied fractional calculus in cancer research to observe the memory effect of fractional operators on tumor-immune interaction and cancer treatments. An optimal combination of cancer treatment strategies for a Caputo fractional cancer treatment model has been proposed in [21] to examine the effect of obesity and the fractional order on the system. Sohail et al. [22] explored the effect of fractional order and the antigenicity of the tumor on the tumor-immune system. In [23], the memory effect of fractional derivatives was observed in a fractional-order cancer model, which described the tumor-immune interaction in the presence of ACI and IL-2 therapy. Baleanu et al. [24] analyze the tumor-immune surveillance mechanism by considering a fractional-order cancer model of singular and non-singular operators. They have also investigated the efficacy of optimally administered chemotherapy treatment in the proposed model. In [25], a radiotherapy procedure was applied to a fractional-order cancer treatment model and showed the effect of radiotherapy on tumors and normal cells.

The role of the Caputo fractional operator on the stability and dynamics of the tumor-macrophage system has been investigated in [26]. In [27], the author observed how the different fractional derivatives in both fractional and fractal-fractional senses increase the complexity of tumor-immune mechanisms to confirm the memory effect of the fractional operator. A comparative study between Caputo and Caputo Fabrizio fractional operators has been performed by analyzing a fractional order tumor-immune model consisting of IL-2 cytokines and an anti-PD-L1 inhibitor [28]. The role of macrophages on tumor-immune interaction and the effect of the fractional operator were observed in [29] by studying a Caputo fractional lung cancer model and fitting it with real data. The dynamics of tumor-immune interaction were observed in [30] by varying the Atanagan-Baleanu fractal-fractional derivative. A breast cancer fractional order model has been analyzed using the Levenberg-Marquardt backpropagation scheme (LMBS) and neural networks to explore the impact of immune-chemotherapeutic treatment [31]. A fractal-fractional operator cancer model has been discussed in [32] to identify the association among the cancer cells, immune system, and anti-PD-L1 inhibitor and the effect of fractional parameters on the model. The literature reviewed above inspired this study to propose a fractional-order cancer treatment model. We modify the model proposed in [8] by using the Caputo fractional operator to investigate the effect of optimally administered chemotherapy and stem cell therapy on tumor growth and immune and normal cells. We will also examine the effect of fractional order on the proposed system.

## Basic terminology

In this section, we will introduce the definitions and characteristics of the Caputo fractional derivative [33–36] that are necessary for the sake of formulating our model.

Defining the left Caputo fractional operator for 0 < *α* < 1 as:

ACDtαφ(t)=1Γ(1-α)∫Atφ′(s)(t-s)αds. (1)

The related right Caputo fractional operator can be defined as:

tCDBαφ(t)=-1Γ(1-α)∫tBφ′(s)(s-t)αds. (2)

Additionally, the appropriate Riemann-Liouville differentiation operator can be defined as:

tRLDBαφ(t)=-ddt∫tBφ(s)(s-t)αds. (3)

The corresponding fractional integral is

AItαφ(t)=1Γ(α)∫At(t-s)α-1φ(s)ds. (4)

The relationship between Liouville differentiation operators and the right Caputo fractional operator can be expressed as

tRLDBαφ(t)=tCDBαφ(t)+φ(B)(B-t)-αΓ(1-α). (5)

**Definition 1**. [37, 38] *For α* > 0, *we can define the integration of arbitrary order φ*(*t*) ∈ *L*[0, *T*] *as*

$$\begin{array}{c} I_{+0}^{\alpha }\left[\varphi \left(t\right)\right]=\frac{1}{\Gamma (\alpha )}\int_{0}^{t}{\left(t-s\right)}^{\alpha -1}\varphi \left(s\right)ds. \end{array}$$ (6)

**Definition 2**. [37, 38] *If φ*(*t*) ∈ *C*[0, *T*], *then the Caputo derivative with arbitrary order α* (0 < *α* ≤ 1) *may be defined as*

0CDtαφ(t)={1Γ(1-α)∫0t(t-s)-αφ(t)′(s)ds,0<α<1,dφ(t)dt,α=1, (7)

*assuming that the integral that appears on RHS is pointwise convergence on* (0, ∞).

**Lemma 1**. *Corresponding to the FDE*

0CDtαφ(t)=ϱ(t),0<α≤1, (8)

*the solution is given by*

$$\begin{array}{c} \varphi \left(t\right)=C_{0}+I_{+0}^{\alpha }\varrho \left(t\right),C_{0}\in R. \end{array}$$ (9)

## Model description

This section will formulate a fractional-order cancer treatment model based on the model discussed in [8]. In [8], the author proposed a classical integer order model to explore the efficacy of stem cell therapy, which helps modify the immune system, and chemotherapy in reducing tumor cells. Here, we extend the model [8] to a fractional one by including a normal cell population, which was not considered in [8], to see the effect of given therapies on it. Suppose *T*(*t*), *E*(*t*), *N*(*t*), *S*(*t*), and *M*(*t*) be the densities of tumor cells, effector-immune cells, normal cells, stem cells, and amount of the chemotherapeutic agents at any time *t* > 0, respectively. Then, the fractional-order cancer-treatment model that we considered takes the following form:

0cDtαT(t)=r1αT(1-b1T)-p3αET-kTαMT0cDtαE(t)=ρα-μαE+p1αESS+1-p2α(T+M)E0cDtαN(t)=r2αN(1-b2N)+hαSN-p4αTN-kNαMN0cDtαS(t)=γ1αS-kSαMS0cDtαM(t)=v(t)-γ2αM, (10)

where 0cDtα represents the Caputo fractional operator and *α* ∈ (0, 1). The description of the parameters and their values is given in Table 1. The initial condition of the state variables is considered as

$$\begin{array}{c} T\left(0\right)=T_{0},\;E\left(0\right)=E_{0},\;N\left(0\right)=N_{0},\;S\left(0\right)=S_{0},\;\;M\left(0\right)=M_{0}. \end{array}$$ (11)

**Table 1:** Description and values of the parameters.

Parameter	Description	Value
*r*_1_	Proliferation rate of tumor cells	0.00431 *day*^−1^ [6]
*b*_1_	Reciprocal of tumor cells’ carrying capacity	1.02 × 10^−9^*cells*^−1^ [6]
*p*_1_	Interaction rate of stem cells with effector cells	3.41 × 10^−11^*day*^−1^*cells*^−1^ (assumed)
*p*_2_	Interaction rate of effector cells with tumor cells and chemotherapeutic drug	2 × 10^−11^*day*^−1^*cells*^−1^ (assumed)
*p*_3_	Interaction rate of tumor cells with effector cells	6.41 × 10^−11^*day*^−1^*cells*^−1^ [6]
*p*_4_	Interaction rate of normal cells with tumor cells	3.41 × 10^−11^*day*^−1^*cells*^−1^ (assumed)
*k*_*T*_	Response of tumor cells to chemotherapy drug	0.08 *day*^−1^ [6]
*k*_*N*_	Response of normal cells to chemotherapy drug	2 × 10^−11^*day*^−1^ (assumed)
*h*	Response of normal cells to stem cells	3 × 10^−11^*day*^−1^ (assumed)
*k*_*S*_	Response of stem cells to chemotherapy drug	2 × 10^−11^*day*^−1^ (assumed)
*ρ*	Effector cells’ source rate	0.33 *day*^−1^*cells*^−1^ [6]
*μ*	Mortality rate of effector cells	0.204 *day*^−1^ [6]
*r*_2_	Proliferation rate of normal cells	*r*_1_/2 *day*^−1^ (assumed)
*b*_2_	Reciprocal of normal cells’ carrying capacity	*b*_1_/2 *cells*^−1^ (assumed)
*γ*_1_	Decay rate of stem cells	0.1 *day*^−1^ (assumed)
*v*	The influx of external chemotherapy drug that dependents on the time	1 (assumed)
*γ*_2_	Decay rate of chemotherapeutic drug	1 *day*^−1^ [6]

## Qualitative behavior

In this section, we will investigate some fundamental analyses such as the existence of positive solutions, equilibria, linear stability, and Ulam-Hyers stability of the system (10) corresponding to the initial condition (11).

### Existence of positive solutions

To investigate the positivity of the solutions of system (10) associated with (11), we assume that $R_{+}^{5}=\left\{Y\in R_{+}^{5}|Y\leq 0\right\}$, where *Y* = (*T*, *E*, *N*, *S*, *M*)^*T*^.

**Theorem 1**. *Corresponding to the initial condition* (11), *there exists a unique solution of the system* (10) *in* $R_{+}^{5}$ (10).

*Proof*. We would like to refer to [39] for the existence of a unique solution of the system (10) on (0, ∞). Here, we will prove that the solution of the system (10) is in $R_{+}^{5}$. Since,

D0ctαT=0,D0ctαE=ρα≥0,D0ctαN=0,D0ctαS=0,D0ctαM=vt≥0.

Thus, the solution will remain in $R_{+}^{5}$ such that the vector field points belong in $R_{+}^{5}$ for all hyperplanes bounding the positive hyperoctant.

### Equilibria and linear stability

Here, we will examine the positive equilibria of the system (10) and the conditions that lead to their stability. To do this, we fixed the chemotherapeutic dose as *v*(*t*) = *v*. We obtain the equilibria of the system (10), and their stability is as follows:

- Dead equilibria: The dead equilibria will exist when the normal cell population becomes zero, i.e., *N* = 0, which means that the patient is not alive, so we will not consider all the dead equilibria for further investigation.
- Tumor-free equilibrium: The tumor-free equilibrium $P_{0}^{*}\left(0,E_{0}^{*},N_{0}^{*},S^{*},M^{*}\right)$ is given by
  $$\begin{array}{c} T_{0}^{*}=0,\;E_{0}^{*}=\frac{\rho^{\alpha }}{\mu^{\alpha }+p_{2}^{\alpha }M^{*}},\;N_{0}^{*}=\frac{1}{b_{2}^{\alpha }}-\frac{k_{N}^{\alpha }M^{*}}{r_{2}^{\alpha }b_{2}^{\alpha }},\;S^{*}=0,\;M^{*}=\frac{v^{\alpha }}{\gamma_{2}^{\alpha }}. \end{array}$$ (12)
  Clearly, $E_{0}^{*}>0$ and M* > 0; thus, the equilibrium $P_{0}^{*}$ exists if $N_{0}^{*}>0\Rightarrow \frac{1}{b_{2}^{\alpha }}-\frac{k_{N}^{\alpha }M^{*}}{r_{2}^{\alpha }b_{2}^{\alpha }}>0\Rightarrow r_{2}^{\alpha }>k_{N}^{\alpha }\frac{v^{\alpha }}{\gamma_{2}^{\alpha }}$ Thus, the tumor-free equilibrium will exist if the proliferation rate of normal cells is greater than a threshold value $k_{N}^{\alpha }\frac{v^{\alpha }}{\gamma_{2}^{\alpha }}$.
- Co-existing equilibrium: The co-existing equilibrium $P_{1}^{*}\left(T_{1}^{*},E_{1}^{*},N_{1}^{*},S^{*},M^{*}\right)$ is obtained as
  $$\begin{array}{c} \begin{array}{cc} T_{1}^{*} & =\frac{1}{b_{1}^{\alpha }}-\frac{p_{3}^{\alpha }E^{*}+k_{T}^{\alpha }M^{*}}{r_{1}^{\alpha }b_{1}^{\alpha }},\\ E_{1}^{*} & =\frac{\rho^{\alpha }}{\mu +p_{2}\left(T^{*}+M^{*}\right)},\\ N_{1}^{*} & =\frac{1}{b_{2}^{\alpha }}-\frac{p_{4}^{\alpha }T^{*}+k_{N}^{\alpha }M^{*}}{r_{2}^{\alpha }b_{2}^{\alpha }},\\ S^{*} & =0,\\ M^{*} & =\frac{v^{\alpha }}{\gamma_{2}^{\alpha }}. \end{array} \end{array}$$ (13)
  Clearly, $E_{1}^{*}>0$ and M* > 0. Thus, the co-existing equilibrium will exist if $T_{1}^{*}>0$ and $N_{1}^{*}>0$; which implies $r_{1}^{\alpha }>p_{3}^{\alpha }E_{1}^{*}+k_{T}^{\alpha }M^{*}$, $r_{2}^{\alpha }>p_{4}^{\alpha }T_{1}^{*}+k_{N}^{\alpha }M^{*}$. Therefore, if the proliferation rate of tumor cells and normal cells is greater than a threshold value of $p_{3}^{\alpha }E_{1}^{*}+k_{T}^{\alpha }M^{*}$, and $p_{4}^{\alpha }T_{1}^{*}+k_{N}^{\alpha }M^{*}$ respectively, then the co-existing equilibrium will exist.

Now, we will analyze the linear stability of the system (10) at the tumor-free and co-existing equilibrium points. To do this, we stated the following theorems:

**Theorem 2**. *The tumor-free equilibrium* $P_{0}^{*}=\left(0,N_{0}^{*},E_{0}^{*},0,M^{*}\right)$ *will be locally asymptotically stable provided*

$$\begin{array}{c} k_{s}^{\alpha }M^{*}>\gamma_{1}^{\alpha },\\ 2r_{2}^{\alpha }b_{2}^{\alpha }N_{0}^{*}+k_{N}^{\alpha }M^{*}>r_{2}^{\alpha },\\ p_{3}^{\alpha }E_{0}^{*}+k_{T}^{\alpha }M^{*}>r_{1}^{\alpha }. \end{array}$$ (14)

*Proof*. At the tumor-free equilibrium $P_{0}^{*}\left(0,N_{0}^{*},E_{0}^{*},S^{*},M^{*}\right)$, the Jacobian matrix is given by

$$\begin{array}{c} J\left(P_{0}^{*}\right)=[\begin{array}{ccccc} f_{11} & 0 & 0 & 0 & 0\\ f_{21} & f_{22} & 0 & f_{24} & f_{25}\\ f_{31} & 0 & f_{33} & f_{34} & f_{35}\\ 0 & 0 & 0 & f_{44} & 0\\ 0 & 0 & 0 & 0 & f_{55} \end{array}], \end{array}$$ (15)

where

$$\begin{array}{ccc} f_{11} & = & r_{1}^{\alpha }-p_{3}^{\alpha }E_{0}^{*}-k_{T}^{\alpha }M^{*},\;f_{21}=-p_{2}^{\alpha }E_{0}^{*},\\ f_{22} & = & -\mu^{\alpha }-p_{2}^{\alpha }M^{*},\;f_{24}=p_{1}^{\alpha }E_{0}^{*},\;f_{25}=-p_{2}^{\alpha }E_{0}^{*},\\ f_{31} & = & -p_{4}^{\alpha }N_{0}^{*},\;f_{33}=-r_{2}^{\alpha }b_{2}^{\alpha }N_{0}^{*}+r_{2}^{\alpha }\left(-b_{2}^{\alpha }N_{0}^{*}+1\right)-k_{N}^{*}M^{*},\\ f_{34} & = & h^{\alpha }N_{0}^{*},\;f_{35}=-k_{N}^{\alpha }N_{0}^{*},\;f_{44}=-k_{s}^{\alpha }M^{*}+\gamma_{1}^{\alpha },\;f_{55}=-\gamma_{2}^{\alpha }. \end{array}$$

The eigenvalues associated with the Jacobian matrix (15) are

$$\begin{array}{ccc} \lambda_{1} & = & -\gamma_{2}^{\alpha }<0,\\ \lambda_{2} & = & -k_{s}^{\alpha }M^{*}+\gamma_{1}^{\alpha },\\ \lambda_{3} & = & r_{2}-2r_{2}^{\alpha }b_{2}^{\alpha }N_{0}^{*}-k_{N}^{\alpha }M^{*},\\ \lambda_{4} & = & -\mu^{\alpha }-p_{2}^{\alpha }M^{*}<0,\\ \lambda_{5} & = & r_{1}^{\alpha }-p_{3}^{\alpha }E_{0}^{*}-k_{T}^{\alpha }M^{*}. \end{array}$$

Therefore, the tumor-free equilibrium $P_{0}^{*}$ will be locally asymptotically stable if λ_2_ < 0, λ_3_ < 0 and λ_5_ < 0, which gives

$$\begin{array}{c} \begin{array}{cc} k_{s}^{\alpha }M^{*} & >\gamma_{1}^{\alpha },\\ 2r_{2}^{\alpha }b_{2}^{\alpha }N_{0}^{*}+k_{N}^{\alpha }M^{*} & >r_{2}^{\alpha },\\ p_{3}^{\alpha }E_{0}^{*}+k_{T}^{\alpha }M^{*} & >r_{1}^{\alpha }. \end{array} \end{array}$$

Thus, the theorem (2) suggests that the tumor-free equilibrium will be locally asymptotically stable if the decay rate of stem cells, the proliferation rate of normal cells, and the proliferation rate of tumor cells should be less than $k_{s}^{\alpha }M^{*}$, $2r_{2}^{\alpha }b_{2}^{\alpha }N_{0}^{*}+k_{N}^{\alpha }M^{*}$, and $p_{3}^{\alpha }E_{0}^{*}+k_{T}^{\alpha }M^{*}$, respectively.

**Theorem 3**. *The co-existing equilibrium* $P_{1}^{*}=\left(T_{1}^{*},E_{1}^{*},N_{1}^{*},S^{*},M^{*}\right)$ *of the system* (10) *will be locally asymptotically stable if*

$$\begin{array}{c} r_{1}^{\alpha }<k_{T}^{\alpha }M^{*}-p_{3}^{\alpha }E^{*}-2b_{1}^{\alpha }r_{1}^{\alpha }T_{1}^{*},\\ r_{2}^{\alpha }<k_{N}^{\alpha }M^{*}+2b_{2}^{\alpha }r_{2}^{\alpha }N_{1}^{*}+p_{4}^{\alpha }T_{1}^{*},\\ \gamma_{1}^{\alpha }<k_{S}^{\alpha }M^{*}, \end{array}$$ (16)

*and*

$$\begin{array}{c} |arg\left(\lambda_{i}\right)|>\frac{\alpha \pi }{2},i=1,\ldots ,5, \end{array}$$ (17)

*where* λ_*i*_ *are the eigenvalues of the Jacobian matrix evaluated at* $P_{1}^{*}$.

*Proof*. The Jacobian at $P_{1}^{*}$ = $\left(T_{1}^{*},E_{1}^{*},N_{1}^{*},S^{*},M^{*}\right)$ is

$$\begin{array}{c} J\left(P_{1}^{*}\right)=[\begin{array}{ccccc} e_{11} & e_{12} & 0 & 0 & e_{15}\\ e_{21} & e_{22} & 0 & e_{24} & e_{25}\\ e_{31} & 0 & e_{33} & e_{34} & e_{35}\\ 0 & 0 & 0 & e_{44} & 0\\ 0 & 0 & 0 & 0 & e_{55} \end{array}], \end{array}$$ (18)

where

$$\begin{array}{ccc} e_{11} & = & r_{1}^{\alpha }-2r_{1}^{\alpha }b_{1}^{\alpha }T_{1}^{*}-p_{3}^{\alpha }E_{1}^{*}-k_{T}^{\alpha }M^{*},\\ e_{12} & = & -p_{3}^{\alpha }T_{1}^{*},\;e_{15}=-k_{T}^{\alpha }T_{1}^{*},\;e_{21}=-p_{2}^{\alpha }E_{1}^{*}.\\ e_{22} & = & -\mu^{\alpha }-p_{2}^{\alpha }\left(M^{*}+T_{1}^{*}\right),\;e_{24}=p_{1}^{\alpha }E_{1}^{*},\;e_{25}=-p_{2}^{\alpha }E_{1}^{*},\\ e_{31} & = & -p_{4}^{\alpha }N_{1}^{*},\;e_{33}=r_{2}^{\alpha }-k_{N}^{\alpha }M^{*}-2b_{2}^{\alpha }r_{2}^{\alpha }N_{1}^{*}-p_{4}^{\alpha }T_{1}^{*},\\ e_{34} & = & h^{\alpha }N_{1}^{*},\;e_{35}=-k_{N}^{\alpha }N_{1}^{*},\;e_{44}=\gamma_{1}^{\alpha }-k_{S}^{\alpha }M^{*},\;e_{55}=-\gamma_{2}^{\alpha }. \end{array}$$

The eigenvalues of $J(P_{1}^{*})$ are the zeroes of the following polynomial:

$$\begin{array}{c} P\left(\lambda \right)=\left(-\lambda +e_{33}\right)\left(-\lambda +e_{44}\right)\left(-\lambda +e_{55}\right)\left(\lambda^{2}+c_{1}\lambda +c_{2}\right), \end{array}$$ (19)

where *c*_1_ = −(*e*_11_ + *e*_22_) > 0 by using (16) and *c*_2_ = *e*_11_*e*_22_ − *e*_12_*e*_21_.

The eigenvalues corresponding to the Jacobian (18) are

$$\begin{array}{ccc} \lambda_{1} & = & e_{33}=r_{2}^{\alpha }-k_{N}^{\alpha }M^{*}-2b_{2}^{\alpha }r_{2}^{\alpha }N_{1}^{*}-p_{4}^{\alpha }T_{1}^{*}<0,\\ \lambda_{2} & = & e_{44}=\gamma_{1}^{\alpha }-k_{S}^{\alpha }M^{*}<0,\\ \lambda_{3} & = & e_{55}=-\gamma_{2}^{\alpha }<0, \end{array}$$

and

$$\begin{array}{c} \lambda_{4,5}=\frac{-c_{1}\pm \sqrt{c_{1}^{2}-4c_{2}}}{2}. \end{array}$$

Using conditions (16), clearly λ_1_ < 0 and λ_2_ < 0. Also, λ_3_ < 0. The remaining two eigenvalues will be the zeroes of the polynomial *w*(λ) = λ^2^ + *c*_1_λ + *c*_2_. The discriminant of *w*(λ) is $D\left(w\right)=c_{1}^{2}-4c_{2}$. Thus, if *c*_1_ > 0 and $c_{1}^{2}>4c_{2}$, then all the eigenvalues of $J(P_{1}^{*})$ are negative, which satisfies the conditions of Matignon (17). Moreover, If *c*_1_ > 0, $c_{1}^{2}<4c_{2}$ and $\alpha <\frac{2}{\pi }tan^{-1}\frac{\sqrt{4c_{2}-c_{1}^{2}}}{c_{1}}$, then all the eigenvalues satisfy the conditions of Matignon (17) which mean that $P_{1}^{*}$ will be locally asymptotically stable, on the contrary, when $\alpha >\frac{2}{\pi }tan^{-1}\frac{\sqrt{4c_{2}-c_{1}^{2}}}{c_{1}}$, the equilibrium $P_{1}^{*}$ will be unstable. Thus, the theorem holds.

### Ulam–Hyers stability

This section will provide the Ulam-Hyers stability of the solutions of the system (10) on the time interval [0, *T*]. We employ Schauder and Banach’s fixed point results [37, 40] to the system (10) to determine the necessary conditions for the existence and uniqueness of the solution of (10). The system (10) with 0 < *α* ≤ 1 is:

0cDtα[T(t)]=f1(t,T,E,N,S,M),0cDtα[E(t)]=f2(t,T,E,N,S,M),0cDtα[N(t)]=f3(t,T,E,N,S,M),0cDtα[S(t)]=f4(t,T,E,N,S,M),0cDtα[M(t)]=f5(t,T,E,N,S,M), (20)

where

$$\begin{array}{c} T\left(0\right)=T_{0},\;E\left(0\right)=E_{0},\;N\left(0\right)=N_{0},\;S\left(0\right)=S_{0},\;\text{and}\;M\left(0\right)=M_{0}. \end{array}$$ (21)

By using lemma (1), the system (20) reduces to

$$\begin{array}{c} \left\{ \begin{array}{c} T\left(t\right)=T_{0}+\frac{1}{\Gamma (\alpha )}\int_{0}^{t}{\left(t-s\right)}^{\alpha -1}f_{1}\left(s,T\left(s\right),E\left(s\right),N\left(s\right),S\left(s\right),M\left(s\right)\right)ds,\\ E\left(t\right)=E_{0}+\frac{1}{\Gamma (\alpha )}\int_{0}^{t}{\left(t-s\right)}^{\alpha -1}f_{2}\left(s,T\left(s\right),E\left(s\right),N\left(s\right),S\left(s\right),M\left(s\right)\right)ds,\\ N\left(t\right)=N_{0}+\frac{1}{\Gamma (\alpha )}\int_{0}^{t}{\left(t-s\right)}^{\alpha -1}f_{3}\left(s,T\left(s\right),E\left(s\right),N\left(s\right),S\left(s\right),M\left(s\right)\right)ds,\\ S\left(t\right)=S_{0}+\frac{1}{\Gamma (\alpha )}\int_{0}^{t}{\left(t-s\right)}^{\alpha -1}f_{4}\left(s,T\left(s\right),E\left(s\right),N\left(s\right),S\left(s\right),M\left(s\right)\right)ds,\\ M\left(t\right)=M_{0}+\frac{1}{\Gamma (\alpha )}\int_{0}^{t}{\left(t-s\right)}^{\alpha -1}f_{5}\left(s,T\left(s\right),E\left(s\right),N\left(s\right),S\left(s\right),M\left(s\right)\right)ds, \end{array}\right. \end{array}$$ (22)

where *t* ∈ [0, *T*] and *T* < ∞.

Then $\varepsilon_{i}=C\left(\left[0,T\right]\times R_{+}^{5},R_{+}\right)$ is a Banach space.

Moreover, $X=\varepsilon_{1}\times \varepsilon_{2}\times \varepsilon_{3}\times \varepsilon_{4}\times \varepsilon_{5}$ is a complete norm space with norm

$$\begin{array}{c} ∥H∥=\underset{t\in [0,T]}{\text{sup}}\left|H\left(t\right)\right|=\underset{t\in [0,T]}{\text{sup}}[|T(t)|+|E(t)|+|N(t)|+|S(t)|+|M(t)|]. \end{array}$$ (23)

Thus, Eq (22) can be written as

$$\begin{array}{c} H\left(t\right)=H_{0}\left(t\right)+\frac{1}{\Gamma (\alpha )}\int_{0}^{t}{\left(t-s\right)}^{\alpha -1}\varrho \left(s,H\left(s\right)\right)ds, \end{array}$$ (24)

where

$$\begin{array}{c} \begin{array}{cc} H(t) & =(T(t),E(t),N(t),S(t),M(t)),\\ H_{0}\left(t\right) & =(T_{0}\left(t\right),E_{0}\left(t\right),N_{0}\left(t\right),S_{0}\left(t\right),M_{0}\left(t\right)), \end{array} \end{array}$$

and

$$\begin{array}{c} \varrho \left(t,H\left(t\right)\right)=\{\begin{array}{c} f_{1}\left(t,T\left(t\right),E\left(t\right),N\left(t\right),S\left(t\right),M\left(t\right)\right),\\ f_{2}\left(t,T\left(t\right),E\left(t\right),N\left(t\right),S\left(t\right),M\left(t\right)\right),\\ f_{3}\left(t,T\left(t\right),E\left(t\right),N\left(t\right),S\left(t\right),M\left(t\right)\right),\\ f_{4}\left(t,T\left(t\right),E\left(t\right),N\left(t\right),S\left(t\right),M\left(t\right)\right),\\ f_{5}\left(t,T\left(t\right),E\left(t\right),N\left(t\right),S\left(t\right),M\left(t\right)\right). \end{array} \end{array}$$ (25)

Now, we consider the following assumptions:

*A1*: For each $H\left(t\right),\bar{H(t)}\in R\times R$, ∃ a constant $\kappa_{\varrho }>0$ such that $\left|\varrho (t,H\left(t\right)-\varrho \left(t,\bar{H(t)}\right)|\leq \kappa_{\varrho }|H\left(t\right)-\bar{H(t)})\right|$.
*A2*: ∃ constants $\sigma_{\varrho }>0$ and $\tau_{\varrho }>0$, with $|\varrho (t,H\left(t\right)|\leq \sigma_{\varrho }|H\left(t\right)|+\tau_{\varrho }.$

**Theorem 4**. *According to the assumptions A2 and continuity of* ϱ, *there will be at least one solution to the system* (10).

*Proof*. Consider K⊂X as a closed set with B={H(t)∈X:∥H∥≤r,r>0}. Let B:K→K be an operator, then according to the Schauder fixed point theorem, (24) can be expressed as

$$\begin{array}{c} B\left(H\left(t\right)\right)=H_{0}\left(t\right)+\frac{1}{\Gamma (\alpha )}\int_{0}^{t}{\left(t-s\right)}^{\alpha -1}\varrho \left(s,H\left(s\right)\right)ds. \end{array}$$ (26)

Now, for each H∈K, we have

$$\begin{array}{c} \begin{array}{cc} \left|B(H(t))\right| & \leq |H_{0}|+\frac{1}{\Gamma (\alpha )}\int_{0}^{t}{\left(t-s\right)}^{\alpha -1}\left|\varrho \left(s,H\left(s\right)\right)\right|ds\\ & \leq |H_{0}|+\frac{1}{\Gamma (\alpha )}\int_{0}^{t}{\left(t-s\right)}^{\alpha -1}[\sigma_{\varrho }\left|H\right|+\tau_{\varrho }]ds\\ & \leq |H_{0}|+\frac{T^{s}}{\Gamma (\alpha +1)}\left[\sigma_{\varrho }r+\tau_{\varrho }\right], \end{array} \end{array}$$ (27)

which yields

$$\begin{array}{c} ∥B(H)∥\leq r. \end{array}$$ (28)

Therefore, H∈K, which implies B(K)⊆K and **B** is bounded.

By assuming *t*_1_ < *t*_2_ ∈ [0, *T*] and taking

$$\begin{array}{c} \begin{array}{cc} |B\left(H\right)\left(t_{2}\right)-B\left(H\right)\left(t_{1}\right)| & =|\frac{1}{\Gamma (\alpha )}\int_{0}^{t_{2}}{\left(t_{2}-s\right)}^{\alpha -1}\varrho \left(s,H\left(s\right)\right)ds-\frac{1}{\Gamma (\alpha )}\int_{0}^{t_{1}}{\left(t_{1}-s\right)}^{\alpha -1}\varrho \left(s,H\left(s\right)\right)ds|\\ & \leq \frac{1}{\Gamma (\alpha )}[\int_{0}^{t_{1}}[{\left(t_{1}-s\right)}^{\alpha -1}-{\left(t_{2}-s\right)}^{\alpha -1}]\left|\varrho \left(s,H\left(s\right)\right)\right|ds\\ & +\int_{t_{1}}^{t_{2}}{\left(t_{2}-s\right)}^{\alpha -1}\left|\varrho \left(s,H\left(s\right)\right)\right|ds]\\ & \leq \frac{\sigma_{\varrho }r+\tau_{\varrho }}{\Gamma (\alpha +1)}[t_{2}^{\alpha }-t_{1}^{\alpha }+2{\left(t_{2}-t_{1}\right)}^{\alpha }]\to 0\;\text{when}\;t_{1}\to t_{2}. \end{array} \end{array}$$ (29)

Accordingly

$$\begin{array}{c} ∥B\left(H\left(t_{2}\right)\right)-B\left(H\left(t_{1}\right)\right)∥\leq r\to 0\;\text{when}\;t_{1}\to t_{2}. \end{array}$$ (30)

Thus, **B** is the operator of equicontinuous. So, the system (10) has at least one solution.

Now, we will derive the result for the uniqueness of the solution of the system (10).

**Theorem 5**. *The model* (10) *possesses a unique solution if the assumptions A2 and* $\frac{T^{\alpha }}{\Gamma (\alpha +1)}\kappa_{\varrho }<1$ *hold*.

*Proof*. Suppose the operator B:X→X and $H,\bar{H}\in X$, then

$$\begin{array}{c} \begin{array}{cc} ∥B\left(H\right)-B\left(\bar{H}\right)∥ & =\underset{t\in [0,T]}{\text{sup}}|\frac{1}{\Gamma (\alpha )}\int_{0}^{t}{\left(t-s\right)}^{\alpha -1}\varrho \left(s,H\left(s\right)\right)ds\\ & -\frac{1}{\Gamma (\alpha )}\int_{0}^{t}{\left(t-s\right)}^{\alpha -1}\varrho \left(s,\bar{H}\left(t\right)\right)ds|\\ & \leq \frac{T^{\alpha }}{\Gamma (\alpha +1)}\kappa_{\varrho }∥H-\bar{H}∥. \end{array} \end{array}$$ (31)

Now, from (31), we obtain

$$\begin{array}{c} ∥B\left(H\right)-B\left(\bar{H}\right)∥\leq \frac{T^{\alpha }}{\Gamma (\alpha +1)}\kappa_{\varrho }∥H-\bar{H}∥. \end{array}$$ (32)

Thus, **B** is a contraction mapping, and so by using the Banach theorem, the system (10) possesses a unique solution.

**Lemma 2**. *Let z* ∈ *C*[0, *T*] *with z*(0) = 0 *is independent of* H *such that* |*f*(*t*)| ≤ *δ*, *for δ* > 0 *and* 0cDtαH(t)=ϱ(t,H(t))+z(t). *Then the solution of the system* (10)

$$\begin{array}{c} \begin{array}{c} c_{0}D_{t}^{\alpha }H=\varrho (t,H)+z(t),H(0)=H_{0}, \end{array} \end{array}$$ (33)

*satisfies the relation*

$$\begin{array}{c} \begin{array}{ccc} \left|H\left(t\right)-(H_{0}\left(t\right)+\frac{1}{\Gamma (\alpha )}\int_{0}^{t}{\left(t-s\right)}^{\alpha -1}\varrho \left(s,H\left(s\right)\right)ds)\right| & \leq \frac{T^{\alpha }}{\Gamma (\alpha +1)}\delta & =\Psi_{T,\alpha }\delta . \end{array} \end{array}$$ (34)

**Theorem 6**. *If the assumptions (A1)-(A2) and* (2) *are satisfied and* $\Omega =\frac{T^{\alpha }}{\Gamma (\alpha +1)}\kappa_{\varrho }<1$, *then the solution of the system* (10) *is Ulam-Hyers stable on* [0, *T*].

*Proof*. Suppose the solution $\bar{H}\in \varepsilon$ of (24) is unique, then for any solution H∈ε other than $\bar{H}$, we get

$$\begin{array}{c} \begin{array}{cc} |H\left(t\right)-\bar{H}\left(t\right)| & =|H\left(t\right)-(H_{0}\left(t\right)+\frac{1}{\Gamma (\alpha )}\int_{0}^{t}{\left(t-s\right)}^{\alpha -1}\varrho \left(s,\bar{H}\left(s\right)\right)ds)|\\ & \leq |H\left(t\right)-(H_{0}\left(t\right)+\frac{1}{\Gamma (\alpha )}\int_{0}^{t}{\left(t-s\right)}^{\alpha -1}\varrho \left(s,\bar{H}\left(s\right)\right)ds)|\\ & +|\frac{1}{\Gamma (\alpha )}\int_{0}^{t}{\left(t-s\right)}^{\alpha -1}\varrho \left(s,\bar{H}\left(s\right)\right)ds-\frac{1}{\Gamma (\alpha )}\int_{0}^{t}{\left(t-s\right)}^{\alpha -1}\varrho \left(s,\bar{H}\left(s\right)\right)ds|\\ & \leq \Psi_{T,\alpha \delta }+\Omega ∥H-\bar{H}∥.\;\left[\;\text{using}\;\text{Lemma}\;\text{(}2\text{)}\right] \end{array} \end{array}$$ (35)

From (35), we can conclude that the solution of (10) is Ulam-Hyers stable.

## Fractional optimal control problem

From a biomedical perspective, it is necessary to obtain an optimal treatment schedule while developing a cancer treatment model so that the toxicity of the applied drugs on healthy cells should be minimal and the suppression of tumor cells should be maximum as time increases [41–44]. Thus, here also, we consider a fractional optimal control problem to obtain an optimal treatment strategy for chemo-stem cell therapy to minimize the tumor cells and maximize the immune and normal cells. To discuss the fractional optimal control problem, we have followed the works [21, 24, 45]. The cancer treatment model with chemo-drug control is

0cDtαT=r1αT(1-b1T)-p3αET-kTαMT0cDtαE=ρα-μαE+p1αESS+1-p2α(T+M)E0cDtαN=r2αN(1-b2N)+hαSN-p4αTN-kNαMN0cDtαS=γ1αS-kSαMS0cDtαM=v(t)-γ2αM, (36)

satisfying

$$\begin{array}{c} T\left(0\right)=T_{0},\;E\left(0\right)=E_{0},\;N\left(0\right)=N_{0},\;S\left(0\right)=S_{0},\;\text{and}\;M\left(0\right)=0\;\text{if}\;v\left(0\right)=0. \end{array}$$ (37)

We define the objective functional as

$$\begin{array}{c} J\left(v\left(t\right)\right)=\int_{0}^{t_{f}}[B_{1}T+\frac{1}{2}B_{2}v^{2}]dt. \end{array}$$ (38)

So, we seek an optimal control v* such that

$$\begin{array}{c} J\left(v^{*}\right)=\text{min}\left\{J\left(v\right):v\in V\right\}, \end{array}$$ (39)

where $V=\{v:\text{v}\;\text{is}\;\text{measurable},0\leq v\left(t\right)\leq 1,t\in \left[0,t_{f}\right]\}$ is the admissible control set.

Now, we will derive the necessary optimality conditions for the optimal control problem (37) and (38).

**Theorem 7**. *Let* (T*, E*, N*, S*, M*) *be the solution of the system* (36) and v* *is the optimal value of v correspond to the functional* (38). *Then, there exist adjoint variables* (λ_1_, λ_2_, λ_3_, λ_4_, λ_5_) *satisfying the FOCP* (37) *with transversality conditions*

$$\begin{array}{c} \lambda_{i}\left(t_{f}\right)=0\;where\;i=1,2,3,4,5. \end{array}$$ (40)

*Also*,

$$\begin{array}{c} v^{*}=\text{min}\left\{max(\frac{-\lambda_{5}}{B_{2}},0),1\right\}. \end{array}$$ (41)

**Proof.** From the Lagrangian definition, we assume the function $H(T,E,N,S,M,\lambda_{i},v)$ where *i* = 1, 2, 3, 4, 5 as follows:

$$\begin{array}{ccc} H(T,E,N,S,M,\lambda_{i},v) & = & B_{1}T+\frac{1}{2}B_{2}v^{2}+\lambda_{1}{(}_{0}^{c}D_{t}^{\alpha }T)+\lambda_{2}{(}_{0}^{c}D_{t}^{\alpha }E)\\ & + & \lambda_{3}{(}_{0}^{c}D_{t}^{\alpha }N)+\lambda_{4}{(}_{0}^{c}D_{t}^{\alpha }S)+\lambda_{5}{(}_{0}^{c}D_{t}^{\alpha }M). \end{array}$$ (42)

Now, differentiating (42) with respect to *T*, *E*, *N*, *S*, and *M*, we get the adjoint variables as:

$$\begin{array}{c} \dot{\lambda_{i}}=-\frac{\partial H}{\partial Y}\;\text{where}\;Y={\left(T,E,N,S,M\right)}^{T}. \end{array}$$ (43)

Thus,

$$\begin{array}{ccc} \dot{\lambda_{1}} & = & -\frac{\partial H}{\partial T}\\ \; & = & -B_{1}-\lambda_{1}\left(p_{3}^{\alpha }E+K_{T}^{\alpha }M+2r_{1}^{\alpha }b_{1}T-r^{\alpha }-r^{\alpha }\right)+\lambda_{2}\left(P_{2}^{\alpha }E\right)+\lambda_{3}\left(P_{4}^{\alpha }\right),\\ \dot{\lambda_{2}} & = & -\frac{\partial H}{\partial E}\\ \; & = & \lambda_{1}\left(p_{3}^{\alpha }T\right)+\lambda_{2}\left(\mu^{\alpha }+p_{2}^{\alpha }\left(T+M\right)-\frac{p_{1}^{\alpha }SE}{S+1}\right)+\lambda_{3}\left(P_{4}^{\alpha }\right),\\ \dot{\lambda_{3}} & = & -\frac{\partial H}{\partial N}\\ \; & = & \lambda_{3}\left(p_{4}^{\alpha }T+k_{N}^{\alpha }M+2b_{2}r_{2}^{\alpha }N-r_{2}^{\alpha }-h^{\alpha }S\right),\\ \dot{\lambda_{4}} & = & -\frac{\partial H}{\partial S}\\ \; & = & \lambda_{2}\left(\frac{p_{1}^{\alpha }E}{{\left(S+1\right)}^{2}}\right)+\lambda_{3}\left(-h^{\alpha }N\right)+\lambda_{4}\left(K_{S}^{\alpha }-\gamma_{1}\right),\\ \dot{\lambda_{5}} & = & -\frac{\partial H}{\partial E}\\ \; & = & \lambda_{1}\left(k_{T}^{\alpha }T\right)+\lambda_{2}\left(p_{2}^{\alpha }E\right)+\lambda_{3}\left(K_{N}^{\alpha }N\right)+\lambda_{4}\left(k_{S}^{\alpha }S\right)+\lambda_{5}\left(\gamma_{2}\right), \end{array}$$ (44)

where λ_*i*_(*t*_*f*_) = 0, *i* = 1, 2, …, 5 are the transeversality conditions.

On minimizing the Lagrangian *H* with respect to the control variable *v* at *v*, we must have

$$\begin{array}{c} \frac{\partial H}{\partial v(t)}=0. \end{array}$$ (45)

Thus, differentiating the Lagrangian (42) with respect to v on the set *V*, we obtain

$$\begin{array}{c} \frac{\partial H}{\partial v(t)}=B_{2}v+\lambda_{5}=0. \end{array}$$ (46)

On solving (46) and assuming $v^{*}=v$, we get

$$\begin{array}{c} v^{*}=-\frac{\lambda_{5}}{B_{2}}. \end{array}$$ (47)

Therefore, v*(*t*) can be expressed as

$$\begin{array}{c} v^{*}\left(t\right)=\text{min}\left\{\text{max}\left(-\frac{\lambda_{5}}{B_{2}},0\right),1\right\}. \end{array}$$ (48)

## Discretization technique

In this section, we will discuss a discretization technique with the help of the work [21] to solve the system (36) numerically. Dividing the interval *I* = (0, *t*_*N*_] into many sub-intervals as 0 = *t*_0_ < *t*_1_ < … < *t*_*N*_ with △*t* = *T*/*N* as step size. Now, we will discretize the left/right Caputo fractional derivative.

Defining the left Caputo fractional derivative as

0cDtαφ(t)=1Γ(1-α)∫0t(t-s)-αφ′(s)ds,0<α<1.

Using the L1 method [46] to discretize the left Caputo fractional derivative is as follows:

Putting *t* = *t*_*n*_ and collecting the yield integrals into one summation, we get

0cDtαφ(tn)=1Γ(1-α)Σj=1n∫tj-1tj(tn-s)-αφ′(s)ds.

Now, we replace the first-order derivative in the above equation by the forward difference quotient $\frac{\varphi \left(t_{j}\right)-\varphi \left(t_{j-1}\right)}{△t}$ and integrating by power rule, we have

0cDtαφ(tn)=-1△t(1-α)Γ(1-α)Σj=1n[φ(tj)-φ(tj-1)][(tn-tj)-α+1-(tn-tj-1)-α+1].

Using the relations *t*_*n*_ = *n*(△ *t*), *t*_*j*_ = *j*(*t*), *t*_*j*−1_ = (*j* − 1) △ *t*, and after few calculations and abbreviations, we reduce

0cDtαφ(tn)=△t-αΓ(2-α)Σj=1n[φ(tj)-φ(tj-1)][(n-j+1)1-α-(n-j)1-α]=B0Σj=1n(φ(tj)-φ(tj-1))(δj,nC), (49)

where $B_{0}=\frac{-△t^{-\alpha }}{\Gamma (2-\alpha )},\;\delta_{j,n}^{C}={\left(n-j\right)}^{1-\alpha }-{\left(n-j+1\right)}^{1-\alpha }$.

We enforce (49) to the state Eq (36), then using the Newton method for 1 ≤ *n* ≤ *N* to the linearized nonlinear state equation, we solve the following system using iteration

$$\begin{array}{c} {}[\begin{array}{ccccc} \delta_{n,n}^{C}B_{0}-q_{11} & -q_{12} & 0 & 0 & -q_{15}\\ -q_{21} & \delta_{n,n}^{C}B_{0}-q_{22} & 0 & -q_{24} & -q_{25}\\ -q_{31} & 0 & \delta_{n,n}^{C}B_{0}-q_{33} & -q_{34} & -q_{35}\\ 0 & 0 & 0 & \delta_{n,n}^{C}B_{0}-q_{44} & -q_{45}\\ 0 & 0 & 0 & 0 & \delta_{n,n}^{C}B_{0}-q_{55} \end{array}][\begin{array}{c} \delta T\\ \delta E\\ \delta N\\ \delta S\\ \delta M \end{array}]=[\begin{array}{c} A_{1}\\ A_{2}\\ A_{3}\\ A_{4}\\ A_{5} \end{array}], \end{array}$$

where

$$\begin{array}{c} \left\{ \begin{array}{cc} q_{11} & =r_{1}^{\alpha }-2r_{1}^{\alpha }b_{1}T_{n}-p_{3}^{\alpha }E_{n}-k_{T}^{\alpha }M_{n},\;q_{12}=-p_{3}^{\alpha }T_{n},\;q_{15}=-k_{T}^{\alpha }M_{n},\\ q_{21} & =-p_{2}^{\alpha }E_{n},\;q_{22}=-\mu^{\alpha }+\frac{p_{1}^{\alpha }S_{n}}{S_{n}+1}-p_{2}^{\alpha }\left(T_{n}+M_{n}\right),\;q_{24}=\frac{p_{1}^{\alpha }E_{n}}{{\left(S_{n}+1\right)}^{2}},\;q_{25}=-p_{2}^{\alpha }E_{n},\\ q_{31} & =-p_{4}^{\alpha }N_{n},\;q_{33}=r_{2}^{\alpha }-2r_{2}^{\alpha }b_{2}N_{n}+h^{\alpha }S_{n}-p_{4}^{\alpha }T_{n}-k_{N}^{\alpha }M_{n},\;q_{34}=h^{\alpha }N_{n},\;q_{35}=-k_{N}^{\alpha }N_{n},\\ q_{44} & =\gamma_{1}^{\alpha }-k_{S}^{\alpha }M_{n},\;q_{45}=-k_{S}^{\alpha }S_{n},\;q_{55}=-\gamma_{2}^{\alpha }, \end{array}\right. \end{array}$$ (50)

and obtain the solution as:

$$\begin{array}{c} \left\{ \begin{array}{cc} A_{1} & {=}_{0}^{c}D_{t}^{\alpha }{T\left(t\right)|}_{t=t_{n}}-\left(r_{1}^{\alpha }T_{n}\left(1-b_{1}T_{n}\right)-p_{3}^{\alpha }E_{n}T_{n}-k_{T}^{\alpha }M_{n}T_{n}\right),\\ A_{2} & {=}_{0}^{c}D_{t}^{\alpha }{E\left(t\right)|}_{t=t_{n}}-\left(\rho^{\alpha }-\mu^{\alpha }E_{n}+\frac{p_{1}^{\alpha }E_{n}S_{n}}{S_{n}+1}-p_{2}^{\alpha }\left(T_{n}+M_{n}\right)E_{n}\right),\\ A_{3} & {=}_{0}^{c}D_{t}^{\alpha }{N\left(t\right)|}_{t=t_{n}}-\left(r_{2}^{\alpha }N_{n}\left(1-b_{2}N_{n}\right)+h^{\alpha }S_{n}N_{n}-p_{4}^{\alpha }T_{n}N_{n}-k_{N}^{\alpha }M_{n}N_{n}\right),\\ A_{4} & {=}_{0}^{c}D_{t}^{\alpha }{S\left(t\right)|}_{t=t_{n}}-\left(\gamma_{1}^{\alpha }S_{n}-k_{S}^{\alpha }M_{n}S_{n}\right),\\ A_{5} & {=}_{0}^{c}D_{t}^{\alpha }{M\left(t\right)|}_{t=t_{n}}-\left(v\left(t\right)-\gamma_{2}^{\alpha }M_{n}\right). \end{array}\right. \end{array}$$ (51)

The discretization of the (right) Caputo fractional derivative 0CDtαφ(t)=−1Γ(1−α)∫ttN(s−t)−αφ′(s)ds,0<α<1, can be found by the previous procedures as:

0CDtαφ(tn)=D0Σj=n+1N(φ(tj-1)-φ(tj))(ξj,nC), (52)

where $D_{0}=-B_{0},\;and\;\xi_{j,n}^{C}=-\delta_{j,n}^{C}={\left(j-n\right)}^{1-\alpha }-{\left(j-n-1\right)}^{1-\alpha }.$

Applying scheme (52) to the adjoint Eq (44) for *N* − 2 ≥ *n* ≥ 0, we get the following system:

$$\begin{array}{c} \begin{array}{cc} & \left[\begin{array}{ccccc} \delta_{n,n+1}^{C}D_{0}-q_{11} & -q_{21} & -q_{31} & 0 & 0\\ -q_{12} & \delta_{n,n+1}^{C}D_{0}-q_{22} & 0 & 0 & 0\\ 0 & 0 & \delta_{n,n+1}^{C}D_{0}-q_{33} & 0 & 0\\ 0 & -q_{24} & -q_{34} & \delta_{n,n+1}^{C}D_{0}-q_{44} & 0\\ -q_{15} & -q_{25} & -q_{35} & -q_{45} & \delta_{n,n+1}^{C}D_{0}-q_{55} \end{array}][\begin{array}{c} {\left(\lambda_{1}\right)}_{n+1}\\ {\left(\lambda_{2}\right)}_{n+1}\\ {\left(\lambda_{3}\right)}_{n+1}\\ {\left(\lambda_{4}\right)}_{n+1}\\ {\left(\lambda_{5}\right)}_{n+1} \end{array}\right]\\ & =[\begin{array}{c} D_{0}\Sigma_{j=n+2}^{N}{\left(\lambda_{1}\right)}_{j}\;\left(\delta_{n,j-1}^{C}-\delta_{n,j}^{C}\right)\\ D_{0}\Sigma_{j=n+2}^{N}{\left(\lambda_{2}\right)}_{j}\;\left(\delta_{n,j-1}^{C}-\delta_{n,j}^{C}\right)\\ D_{0}\Sigma_{j=n+2}^{N}{\left(\lambda_{3}\right)}_{j}\;\left(\delta_{n,j-1}^{C}-\delta_{n,j}^{C}\right)\\ D_{0}\Sigma_{j=n+2}^{N}{\left(\lambda_{4}\right)}_{j}\;\left(\delta_{n,j-1}^{C}-\delta_{n,j}^{C}\right)\\ D_{0}\Sigma_{j=n+2}^{N}{\left(\lambda_{5}\right)}_{j}\;\left(\delta_{n,j-1}^{C}-\delta_{n,j}^{C}\right) \end{array}]. \end{array} \end{array}$$ (53)

## Numerical results

**Fig 1:**
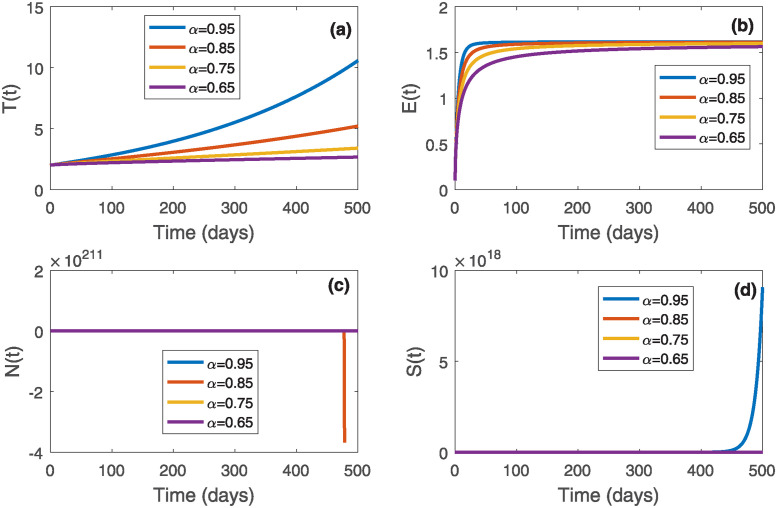
Denisities of (a) Tumor cell population, (b) effector cell population, (c) normal cell population, and (d) stem cell population in the absence of chemotherapy over time.

**Fig 2:**
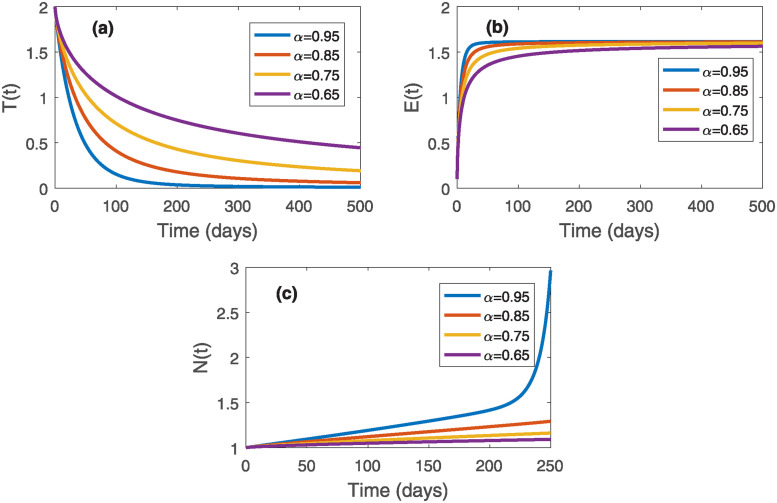
Denisities of (a) Tumor cell population, (b) effector cell population, and (c) normal cell population in the presence of stem cell therapy and chemotherapy over time with a fixed *v*(*t*) = 0.5.

In this section, we will perform a few numerical simulations to validate our analytical findings using MATLAB and the Adam-Bashford-Moulton numerical scheme. We use the parameter values prescribed in Table 1, otherwise stated. The initial conditions of the state variables are taken as *T*_0_ = 2, *E*_0_ = 0.1, *N*_0_ = 1, *M*_0_ = 0.5, and *S*_0_ = 0.5. In addition, we also examine the effect of fractional order *α* on the system (10). To do this, we consider the order of derivatives as *α* ∈ {0.65, 0.75, 0.85, 0.95}. First, we simulate the system (10) without chemotherapeutic drugs, i.e., *M*(*t*) = 0. Then, we found two dead equilibriums as (980392135.34, 1.476, 0, 0), (0, 1.618, 0, 0), and one tumor-free equilibrium as $P_{0}^{*}\left(0,1.618,1960784313.726,0\right)$. Out of them, only the dead equilibrium (980392135.34, 1.476, 0, 0) is stable, which is observed from Fig 1c and 1d; as the normal cell and stem cell population collapse for *α* ∈ {0.65, 0.75, 0.85, 0.95}. Moreover, it can also be noticed that an increase in *α* corresponds to an increase in tumor and effector cell populations. Thus, in the absence of chemotherapy, the effector and stem cell populations cannot eradicate tumors; as a result, normal cell populations die out as time progresses, and patients will not live. Thus, to keep the patients alive, we need to administer external chemotherapeutic drugs with *M*(*t*) ≠ 0 so that the progression of tumor cells can be reduced or stopped. Therefore we solve the system (36) for a fixed *v*(*t*) = 0.5. For this case, there exists one dead equilibrium (0, 1.6177, 0, 0, 0.5) and one tumor-free equilibrium $P_{0}^{*}\left(0,1.618,1960784295.52,0,0.5\right)$. Using Theorem (2) and from Fig 2, it can be observed that the tumor-free equilibrium $P_{0}^{*}\left(0,1.618,1960784295.52,0,0.5\right)$ is stable, i.e., in the presence of chemotherapy, the tumor gets eradicated from the body. Furthermore, it can also be noticed that an increase in *α* corresponds to an increase in normal and effector cell populations, whereas tumor cell populations decrease over time.

Now, to investigate the optimal efficacy of the prescribed treatment strategies, we solve the optimal system (36) for the functional (38) with the initial condition (37) along with the adjoint systems (44) and (48) for *γ*_2_ = 0.1 using the forward-backward sweep method under MATLAB. The initial conditions of the state variables are taken as *T*_0_ = 2, *E*_0_ = 0.1, *N*_0_ = 1, *M*_0_ = 0.5, and *S*_0_ = 0.5. Also, we fix the period of administration of the treatment as 0 to 120 days with the time step size Δ*t* = 0.001. We suppose the optimal value of *v*(*t*) = 0.5. The algorithm used for solving the FOCP is given below:

**Algorithm**

1. Set *ξ* = −1, *δ* = 0.001.
2. Initial the control *v*_*old*_, the state *x*_*old*_ = {*T*_*old*_, *E*_*old*_, *N*_*old*_, *M*_*old*_, *S*_*old*_}, and adjoint *p*_*old*_ = {(λ_*old*_)_1_, (λ_*old*_)_2_, (λ_*old*_)_3_, (λ_*old*_)_4_, (λ_*old*_)_5_}.
3. **while** *ψ* < 0 **do**.
4. Solve the original FOCP (36) for *x* = {*T*, *E*, *N*, *M*, *S*} using *T*_0_, *E*_0_, *N*_0_, *M*_0_, *S*_0_, *v*_0_ forward in time.
5. Solve the adjoint system (44) for *p* = {λ_1_, λ_2_, λ_3_, λ_4_, λ_5_} with λ_*i*_(*t*_*f*_) = 0, *x* = {*T*, *E*, *N*, *M*, *S*} backward in time.
6. Using the Eq (41), update the control to reach *v*(*t*).
7. Compute *χ*_*i*_ = *δ*‖*x*_*i*_‖−‖*x*_*i*_ − (*x*_*old*_)_*i*_‖, *v* = *δ*‖*v*_*j*_‖−‖*v*_*j*_ − (*v*_*old*_)_*j*_‖, *ρ*_*i*_ = *δ*‖*p*_*i*_‖−‖*p*_*i*_ − (*p*_*old*_)_*i*_‖ and calculate *ψ* = *min*{*χ*_*i*_, *v*_*i*_, *ρ*_*i*_} for *i*, *j*.
8. **end while**.

**Fig 3:**
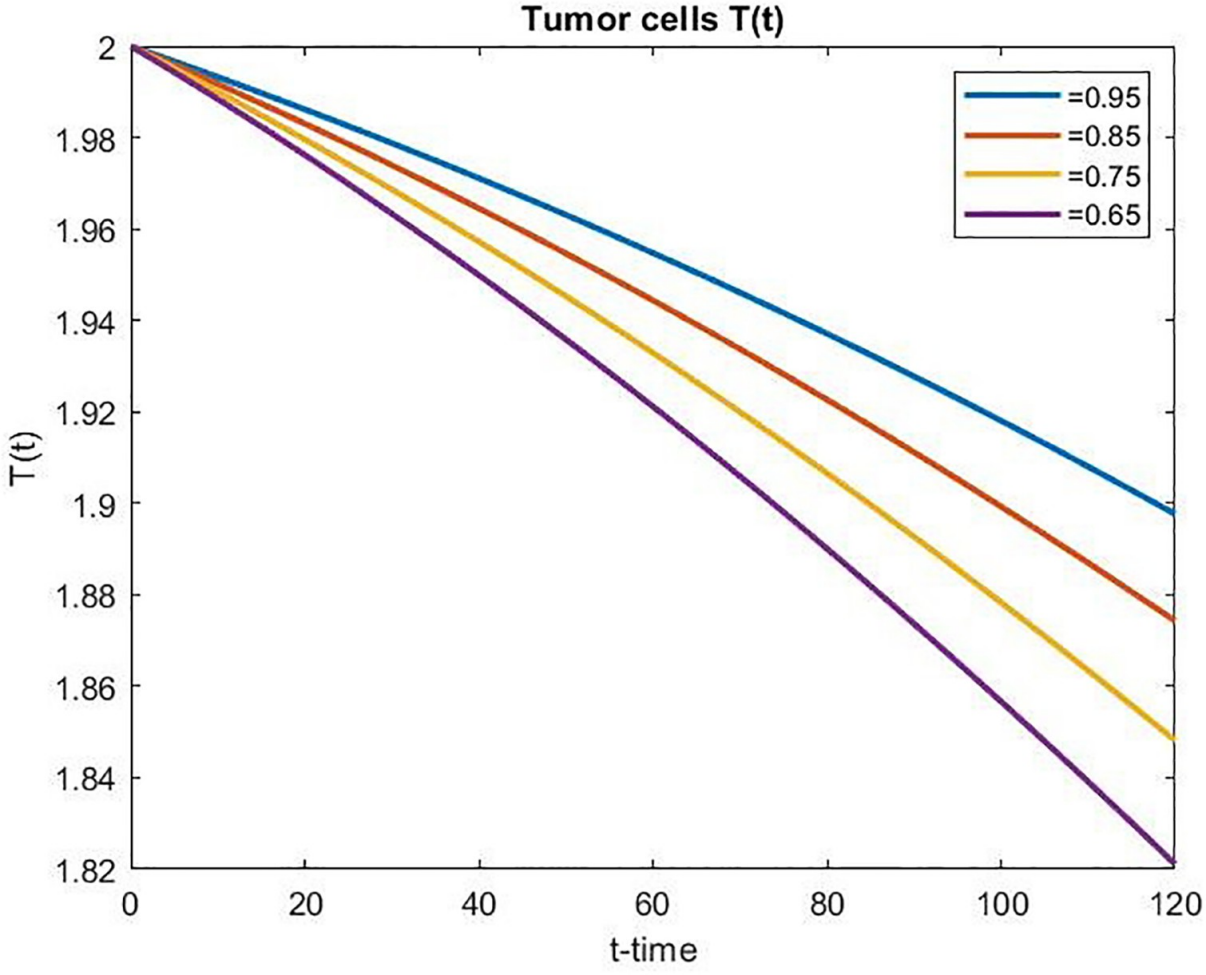
Tumor cell population over time in controlled chemotherapy treatment.

**Fig 4:**
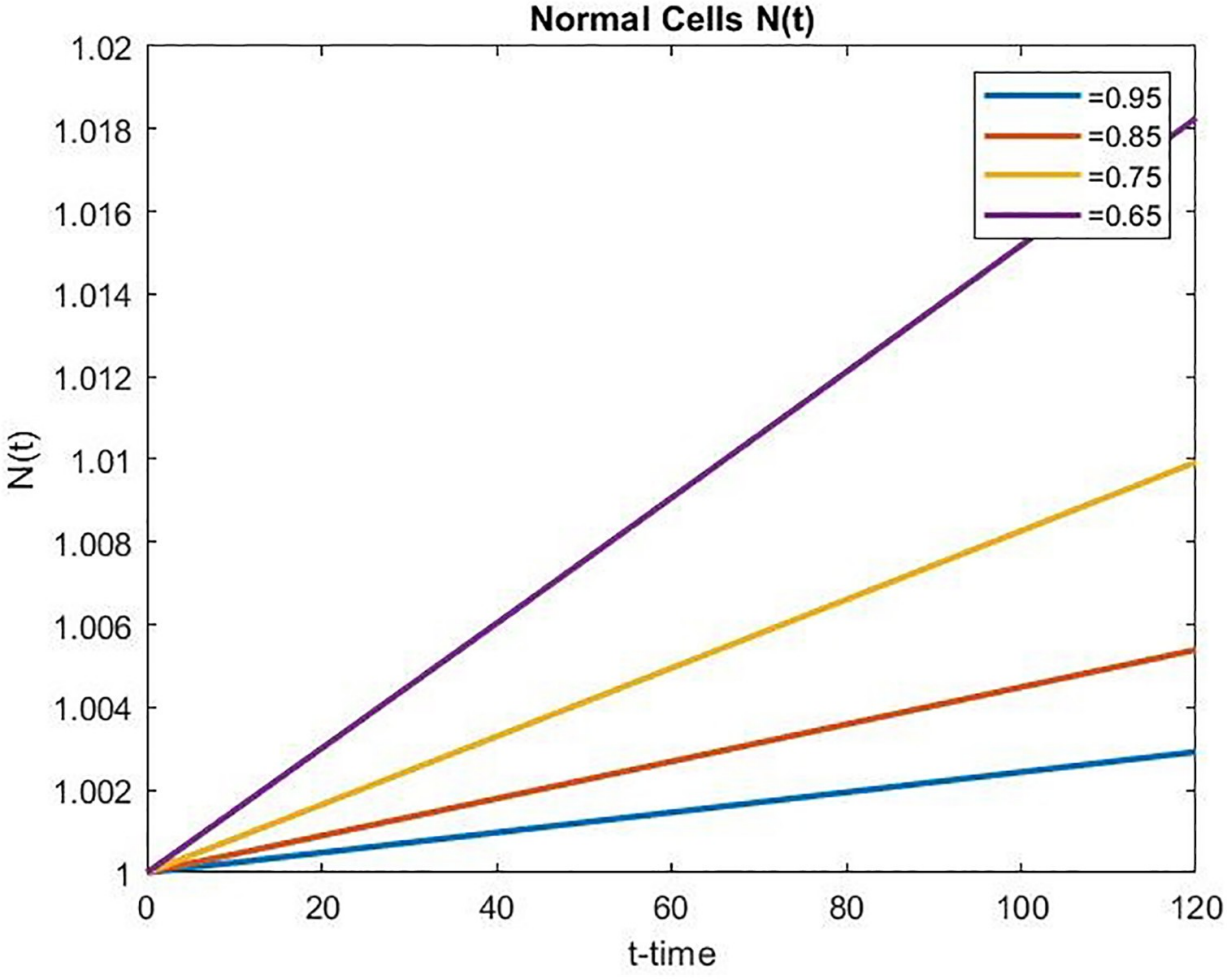
Normal cell population over time in controlled chemotherapy treatment.

**Fig 5:**
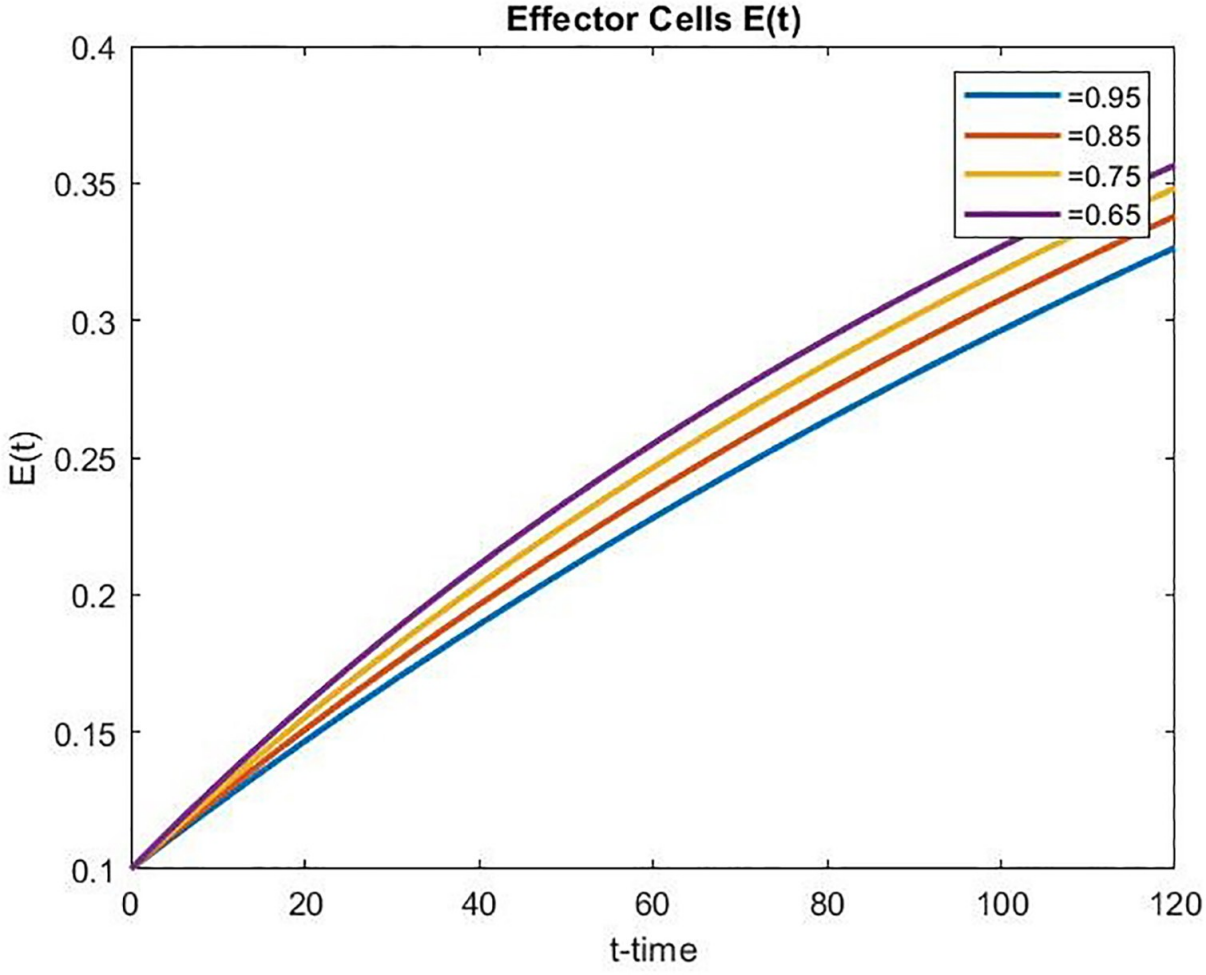
Effector cell population over time in controlled chemotherapy treatment.

We have observed from Figs 3–5 that the number of normal and effector cells increases as time increases, while the tumor cell population is successfully reduced over time. Further, in the case of controlled chemo-drug administration, the number of normal and effector cell populations increased as the fractional order *α* decreased, whereas the number of tumor cell populations decreased as the fractional order *α* decreased.

**Fig 6:**
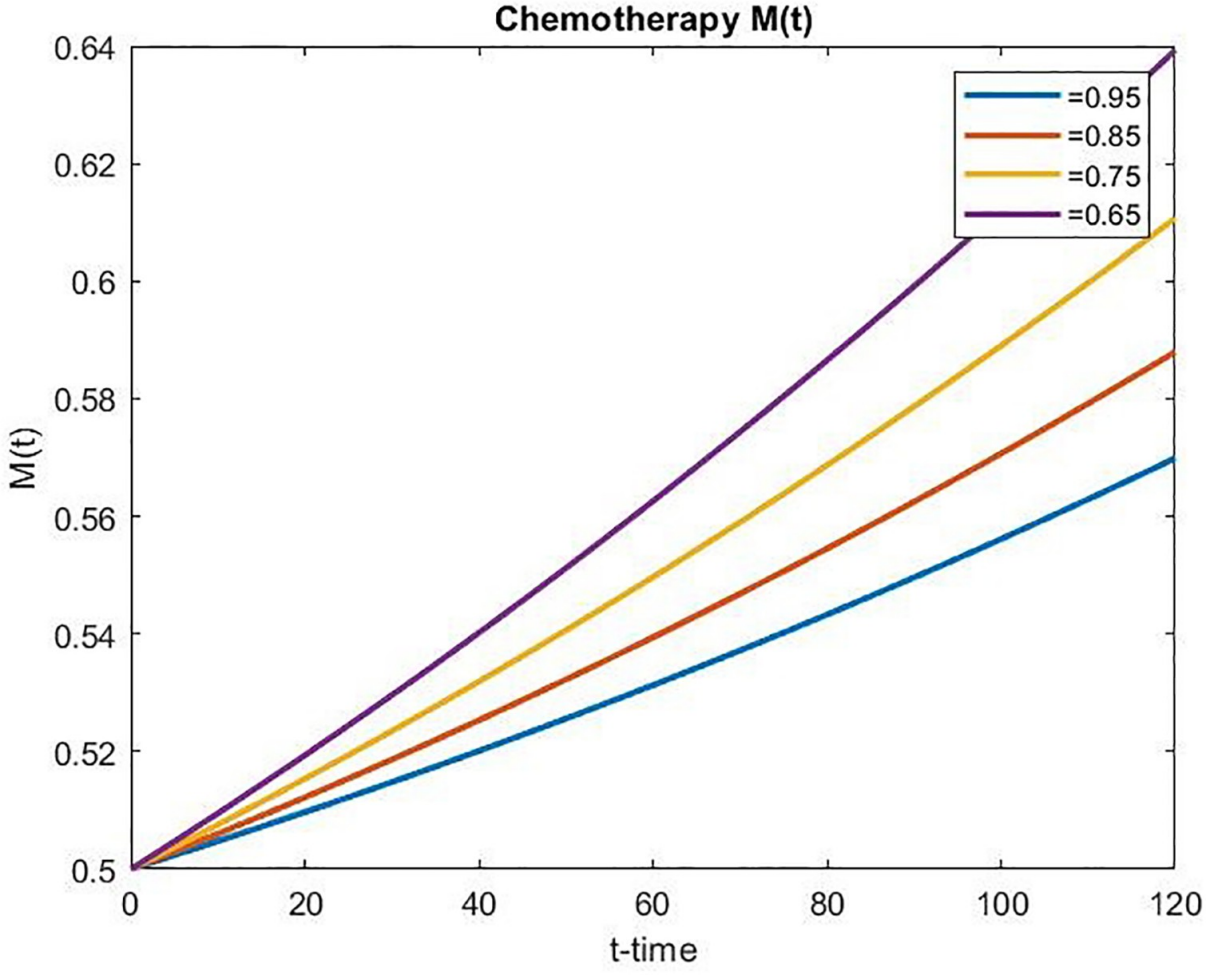
Concentration of chemotherapeutic drug for the uncontrolled case over time.

**Fig 7:**
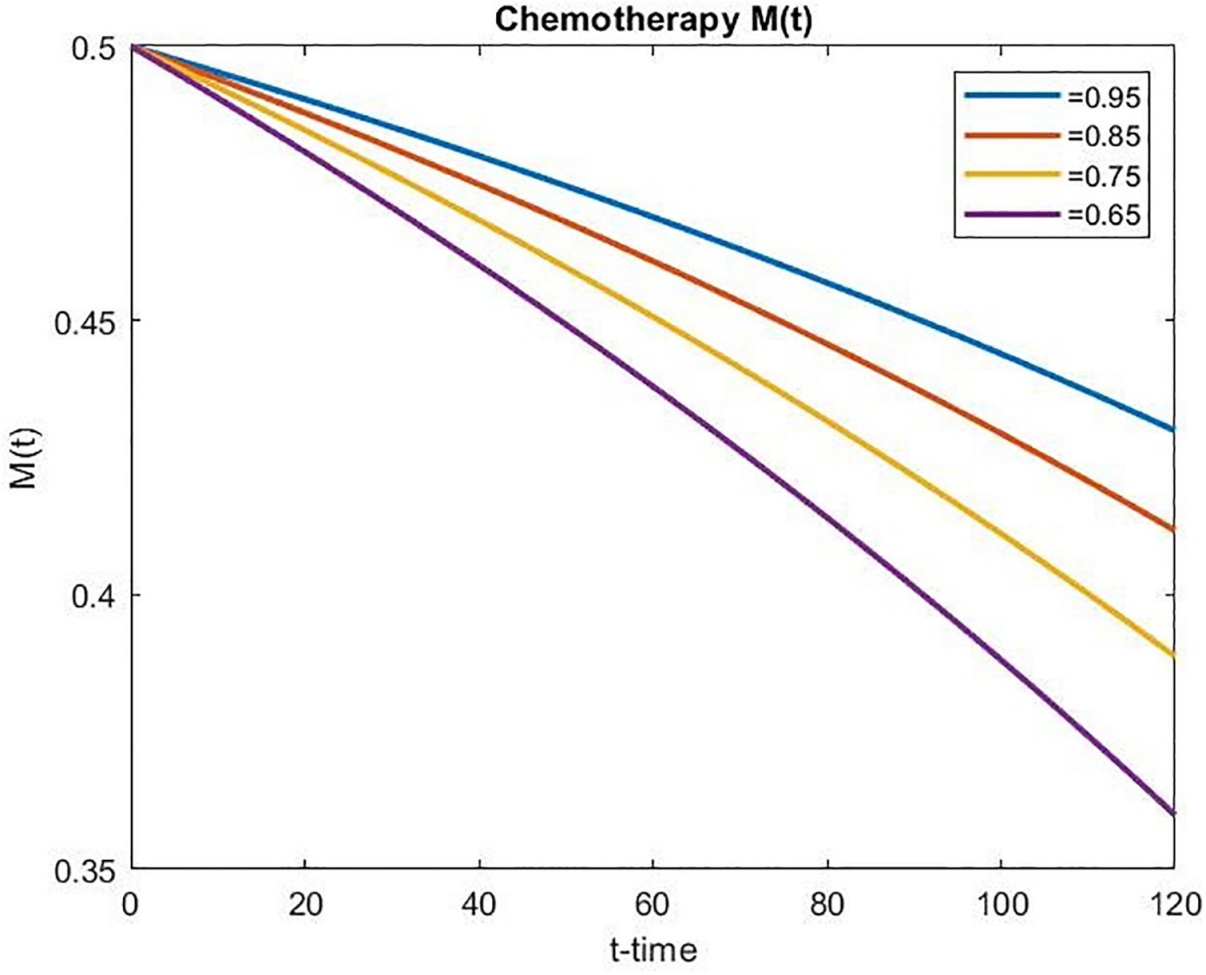
Concentration of chemotherapeutic drug for the controlled case over time.

Figs 6 and 7 present the concentration of chemotherapeutic drugs for the uncontrolled case and the concentration of chemotherapeutic drugs for the controlled case, respectively. In the uncontrolled case, the concentration of chemo-drugs gradually increases, whereas in the controlled case, the concentration of chemo-drugs gradually decreases within the prescribed time duration, which signifies the effect of optimal control treatment. It can also be observed that a decrease in *α* corresponds to an increase in drug concentration in the uncontrolled scenario over time. Conversely, the drug concentration decreases in the controlled scenario as *α* decreases over time. This implies that the functional $J_{\gamma_{1}}$ has been effectively minimized over some time.

**Fig 8:**
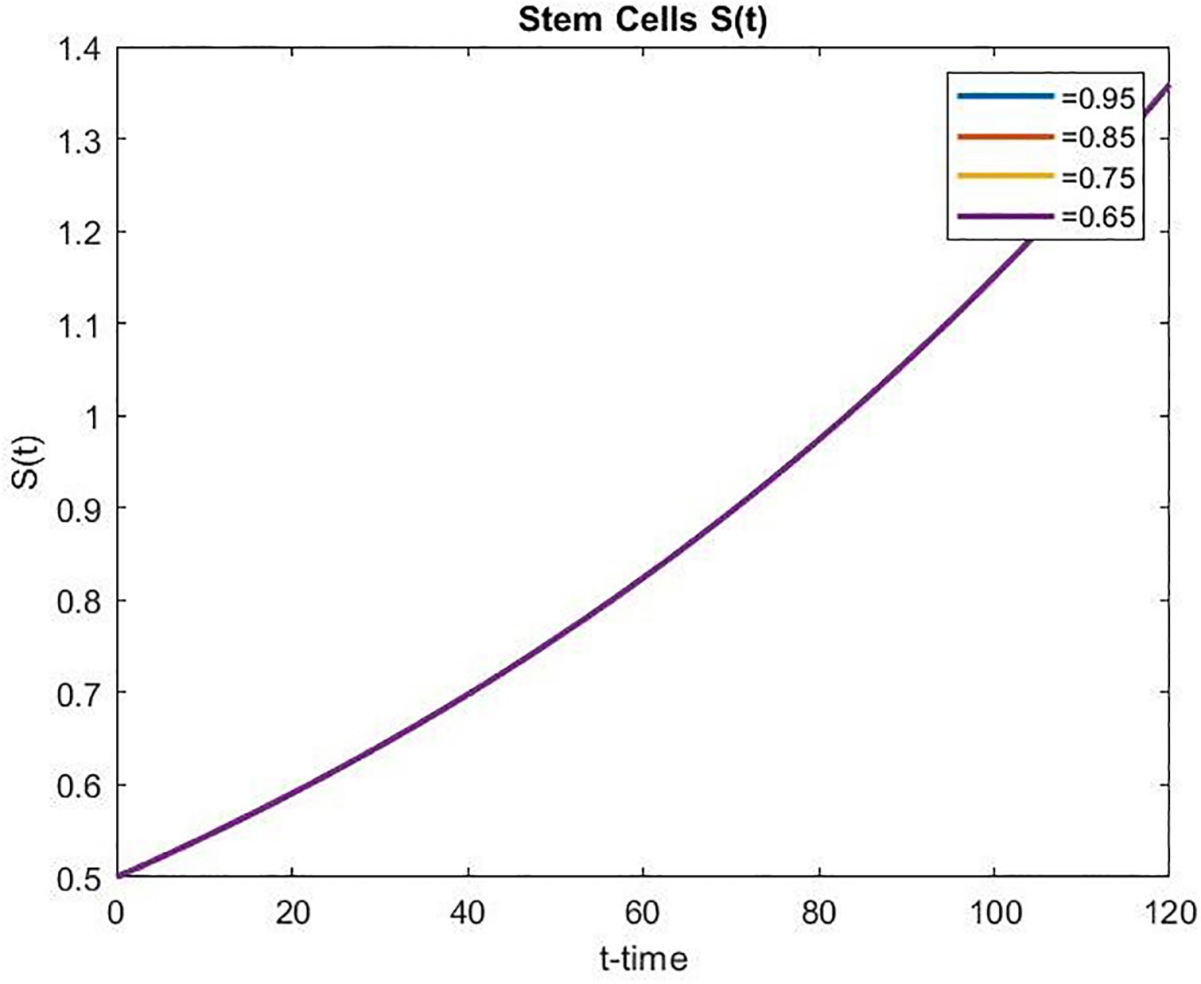
Concentration of stem cells for the uncontrolled case over time.

**Fig 9:**
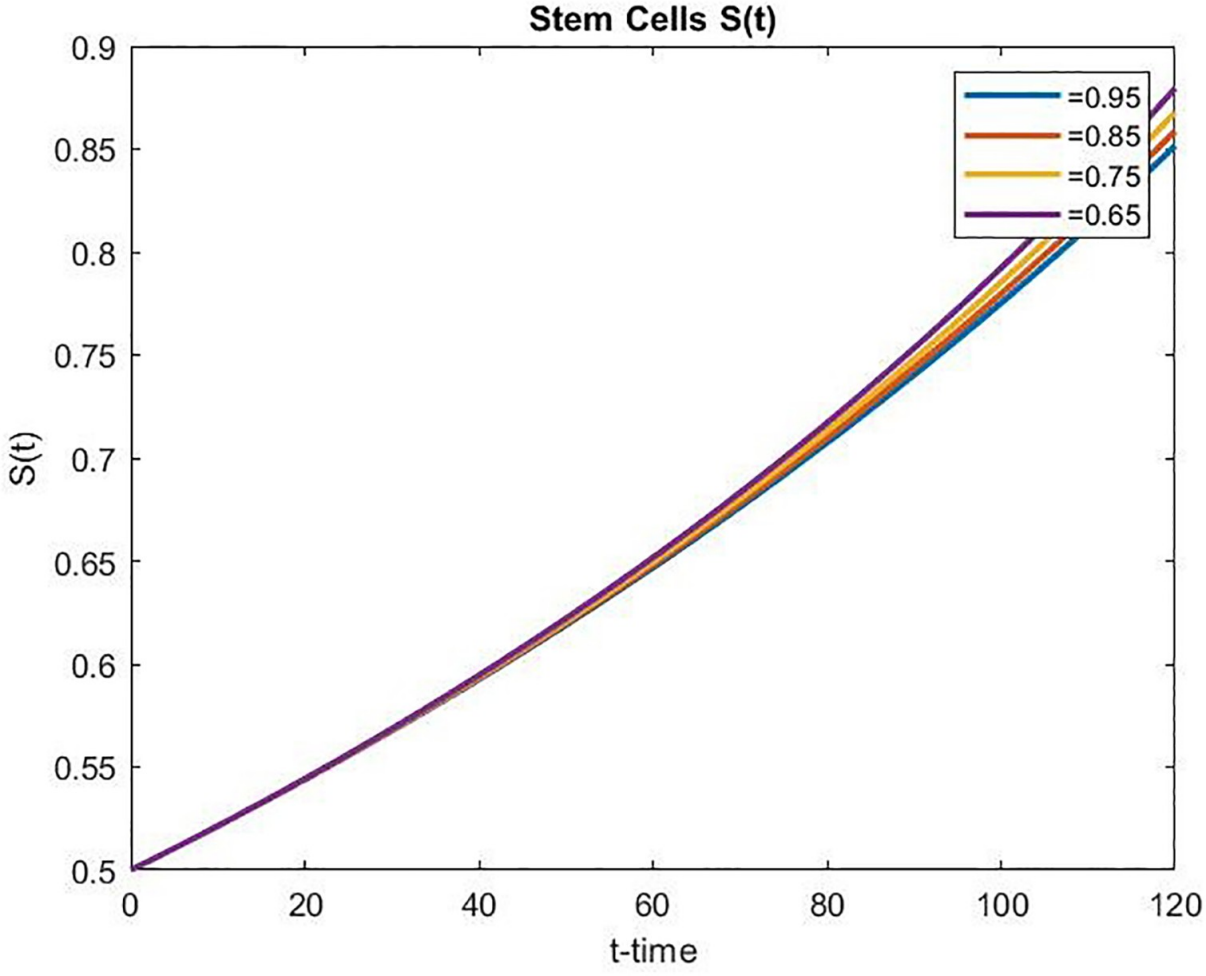
Concentration of stem cells for the controlled case over time.

Figs 8 and 9 present the concentrations of stem cells for the uncontrolled and controlled cases, respectively. As time passes, the number of stem cells increases faster in both uncontrolled and controlled cases, and the increased amount for the uncontrolled case is 44% higher than the controlled case for the same period. Moreover, in the controlled scenario, the stem cell population increases as *α* decreases over time.

The above discussion suggests that the density of tumor cell populations decreases as *α* increases, whereas the density of normal and effector cell populations increases as *α* increases for the uncontrolled administration of both therapies. However, in the controlled administration of both prescribed therapies, a reverse phenomenon is noticed, i.e., the density of tumor cell populations decreases as *α* decreases, whereas the density of normal and effector cell populations increases as *α* decreases. Even in both cases, tumor eradication is possible, but in the controlled one, we require a smaller amount of drug, which may reduce its toxic effect on the patient’s body.

## Conclusion

In this study, we have studied a fractional-order cancer treatment model in the Caputo sense with stem cell therapy and chemotherapy to observe the effect of fractional order. We have examined the existence of positive solutions, equilibria, and their linear stability conditions. We have also investigated the Ulam-Hyers stability of the solutions to the model. Further, we have considered a fractional optimal control problem to obtain an optimal treatment strategy for chemo-stem cell therapy to minimize the tumor cells and maximize the immune and normal cells. Numerical simulation suggests that stem cell therapy and the effector cell cannot reduce or eliminate tumor cells from the body without chemotherapy treatment. However, in controlled chemotherapy cases, the system can eradicate tumor cell populations effectively, so normal and effector cells increase with time. We hope that the findings of this research will help oncologists and medical researchers find an optimal schedule for cancer treatment. This study can be extended by including more cell populations, such as immune macrophages, *T* cells, cytokines, and chemokines responsible for tumor growth and eradication processes. Also, the investigation of the effect of time delay on the response of chemotherapy drugs to the cells and the response of stem cells to the immune system is left as an open research problem in this field.
